# Predicting fault slip via transfer learning

**DOI:** 10.1038/s41467-021-27553-5

**Published:** 2021-12-16

**Authors:** Kun Wang, Christopher W. Johnson, Kane C. Bennett, Paul A. Johnson

**Affiliations:** 1 Geophysics Group, Earth and Environmental Sciences Division, Los Alamos National Laboratory, Los Alamos, NM 87545 USA; 2 Los Alamos National Laboratory, Center for Nonlinear Studies, Los Alamos, NM 87545 USA

**Keywords:** Geophysics, Seismology

## Abstract

Data-driven machine-learning for predicting instantaneous and future fault-slip in laboratory experiments has recently progressed markedly, primarily due to large training data sets. In Earth however, earthquake interevent times range from 10’s-100’s of years and geophysical data typically exist for only a portion of an earthquake cycle. Sparse data presents a serious challenge to training machine learning models for predicting fault slip in Earth. Here we describe a transfer learning approach using numerical simulations to train a convolutional encoder-decoder that predicts fault-slip behavior in laboratory experiments. The model learns a mapping between acoustic emission and fault friction histories from numerical simulations, and generalizes to produce accurate predictions of laboratory fault friction. Notably, the predictions improve by further training the model latent space using only a portion of data from a single laboratory earthquake-cycle. The transfer learning results elucidate the potential of using models trained on numerical simulations and fine-tuned with small geophysical data sets for potential applications to faults in Earth.

## Abstract (web summary)

By teaching machine learning models with earthquake fault numerical simulations laboratory fault slip is predictable. Training the model further with a snippet of laboratory data improves predictions suggesting an approach to probing faults in Earth.

## Introduction

In Earth, predicting instantaneous and future characteristics of fault slip has long been a fundamental goal of geoscientists from an earthquake hazards perspective, but also to improve the basic understanding of fault mechanics^1^. Recent progress towards these goals has been achieved by applying a variety of machine learning (ML) approaches^2–4^ in the laboratory using shear experimental data to describe physical properties^5–11^ and in the Earth using geophysical data to characterize episodic slow-slip that occurs in subduction zones^8,12^, as well as transform faults^13^. In shear experiments, earthquakes or “labquakes”, generated during a single experiment produce a sufficiently large data set for training ML models. However, on natural faults the repeat cycles for all but the smallest earthquakes can span timescales on the order of 10’s-100’s of years. Thus, in-situ geophysical measurements as input for data-driven ML models are generally not available or sufficiently complete for more than a portion of a single earthquake cycle. In particular this problem exists for large magnitude (*M* > 7) earthquakes that produce strong, damaging ground motions. This conundrum presents a serious challenge if the goal is to use data-driven modeling techniques to characterize the physics of fault slip throughout the complete earthquake cycle and to advance earthquake hazards assessment.

Our group’s first work in this subject area^5^ showed that seismic signals emanating from a laboratory fault experiment were rich with information regarding the physics of slip, gleaned from machine learning analysis of the continuous waveform. This led to a large number of complementary efforts by others applying similar approaches to laboratory data, as well as a Kaggle competition on the topic of laboratory earthquake prediction^4^. Subsequently, we showed the same machine learning approach could identify slip characteristics in the Earth using seismic signals broadcast by the slowly-slipping subduction fault in Cascadia^12^ and the San Andreas Fault^13^. The methodology worked for slow slip in Earth because that process exhibits relatively noisy tectonic tremor associated with slip deep on the fault. We have been working on the problem of applying similar approaches to characterize the physics of seismogenic fault slip. To date, these approaches have been challenging, and provide insight regarding how to transition from the laboratory to the Earth. Our belief is that slip rates on natural faults in Earth are so slow that an emitted signal, if it exists, is hidden within cultural and Earth noise that is inherent to continuous seismic recordings. A suite of data-driven models, as applied to Earth, have been unable to tease out a characteristic signal or pattern in the seismic noise. Devising a new approach to characterize fault-slip is the logical next step to overcome these obstacles.

A type of model generalization known as transfer learning^14,15^ is one potential solution to overcome the problem of data sparsity. Generalizing ML models using transfer learning has been applied in a number of areas in geophysics; for instance in seismology applications, transfer learning has been used to improve nonlinear and ill-posed inverse problems associated with seismic imaging, subsurface feature classification, and fault detection^16–20^. We postulate that transfer learning may provide a tractable means of bringing the success of data-driven approaches for predicting fault-slip characteristics in the laboratory to Earth. To our knowledge, no attempt has been made to apply transfer learning using data from numerical simulations to make quantitative predictions of fault slip in laboratory experiments or Earth observations. Herein, we examine the application of such a transfer learning approach to laboratory experiments, which we posit as an important first step in elucidating and laying the foundation for the potential success of applications to Earth.

**Fig. 1:**
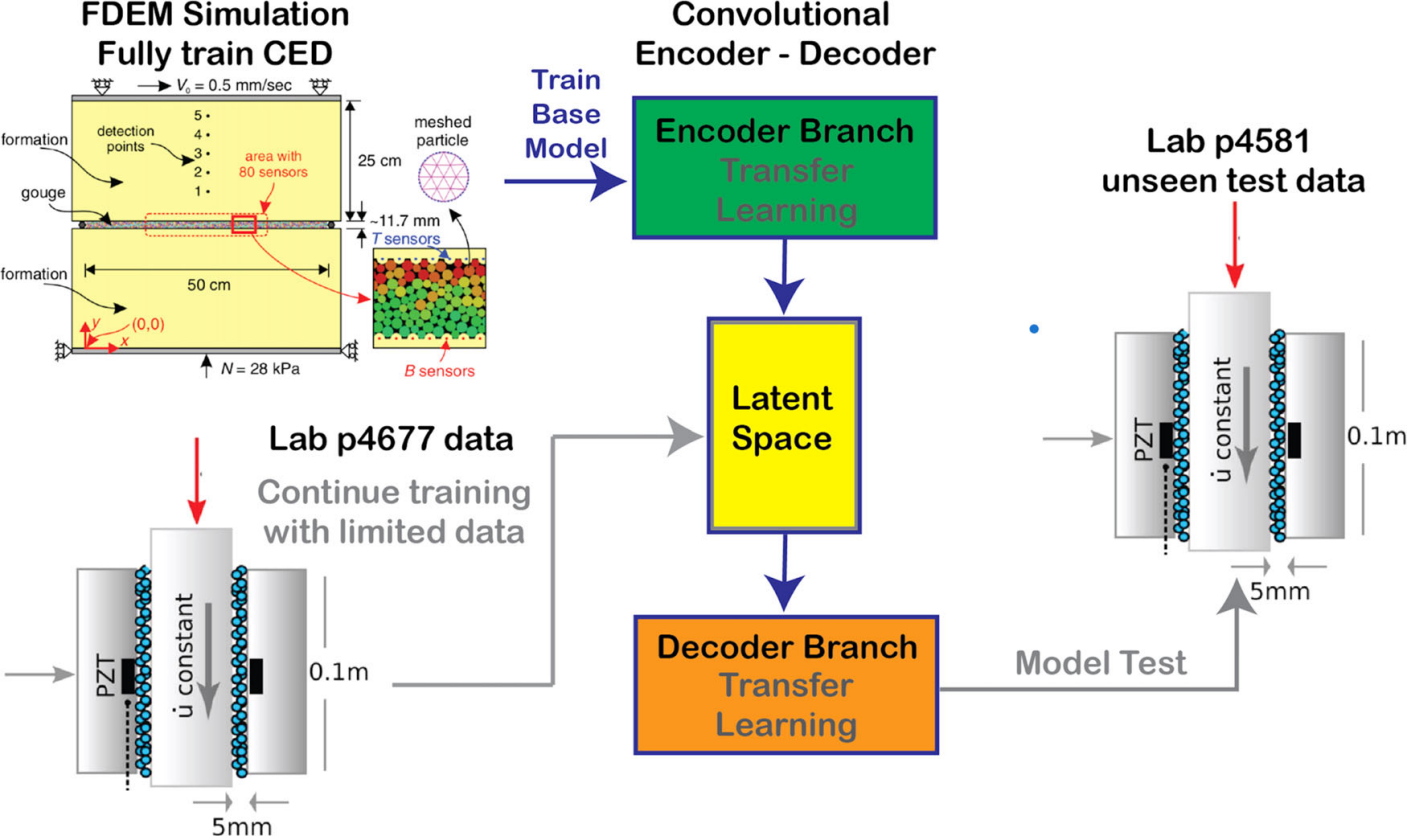
Workflow with numerical simulation (subfigure adapted from ref. ^22^) and experimental configurations (subfigure adapted from ref. ^6^) for the transfer learning analysis. See Methods section for full details about the simulation and experimental data. From the simulation and experiment, characteristics are obtained that include the shear displacement, shear stress, normal stress, bulk friction, kinetic energy (simulation), and acoustic emission (experiment). The simulation kinetic energy is used as a proxy for acoustic emission to train the model to predict the instantaneous bulk friction coefficient at all times throughout the slip cycle. Only the model latent space is then further trained using limited acoustic emission data from the laboratory experiment (number p4677). The simulation-trained encoder and decoder are left unchanged. The new model is used to predict the instantaneous friction for experimental data the model has not previously seen, from a different laboratory experiment (p4581). FDEM finite-discrete element method, CED convolutional encoder-decoder.

In this work, we develop a deep learning convolutional encoder-decoder (CED) model that employs a time-frequency representation of acoustic emissions (AE) from numerical simulations and from laboratory shearing experiments. The model has a U-net architecture that encodes the salient features to a latent space that is then decoded to estimate the instantaneous friction coefficient that evolves through the slip cycle, as measured in the experiment. In brief, the model is initially trained using numerical fault-slip simulation data to learn the mapping between the AE and the friction coefficient. The latent space is then trained using only a small fraction of the laboratory experimental data, and the resulting cross-trained CED model is applied to unseen laboratory experimental data (Fig. 1). If such a procedure works at the laboratory scale, a next step is evaluating a similar approach in Earth, by conducting and applying fault simulations at scale in combination with data from seismogenic faults. In the following, we describe results from the CED model transfer learning and show the successful application of this technique for multiple data sets.

## Results

### Transfer learning from numerical simulations to laboratory shear experiments

**Fig. 2:**
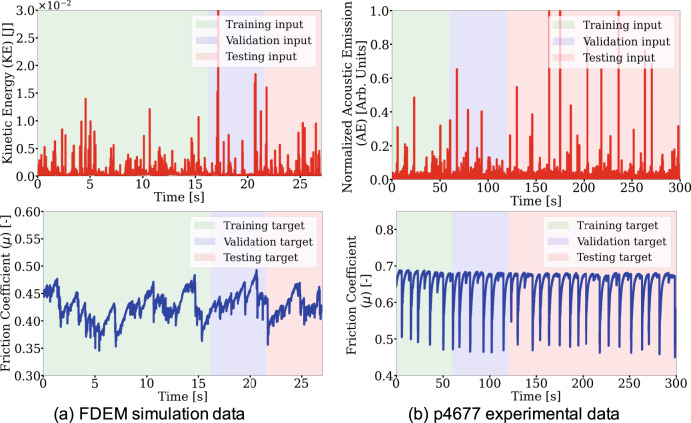
Numerical simulation and experimental data used in the transfer learning analysis. The top row shows the deep learning model input signal as the kinetic energy and acoustic emissions, respectively, and the bottom row shows the target friction coefficient. **a** Finite-discrete element method (FDEM) time series are split into training/validation/testing segments (60/20/20%) shown in green, blue, and pink shades, respectively. The convolutional encoder-decoder is fully trained and tested using these data. **b** The experimental data (p4677) are split into training/validation/testing segments (20/20/60%) to include six cycles of stick-slip events for training the model latent space.

The laboratory data^10,21^ is from a bi-axial shear device that consists of a slider-block bounded by fault gouge and external blocks through which a confining load is applied (Fig. 1). A constant shear velocity is applied and when the system approaches steady state conditions, repetitive stick-slip motion occurs (see “Methods” section and Fig. 2b). The bi-axial device set-up simultaneously measures acoustic emission (AE) and the normal and shear stresses required to calculate the bulk friction coefficient.

The numerical simulation^22^ applies a combined finite-discrete element method (FDEM) model of a fault-shear apparatus resembling the bi-axial device used in the laboratory experiments described here^23^ (see “Methods” section and Fig. 1). The input training data to the CED model from simulation is the kinetic energy, which is a proxy for the measured continuous AE signal in the bi-axial shear experiment. Changes in seismic moment are reflected in variations observed in the kinetic energy; therefore, the kinetic energy represents the kinematic behavior of the granular fault system (see “Methods” section). The CED label data is the bulk friction coefficient between the sliding blocks.

**Fig. 3:**
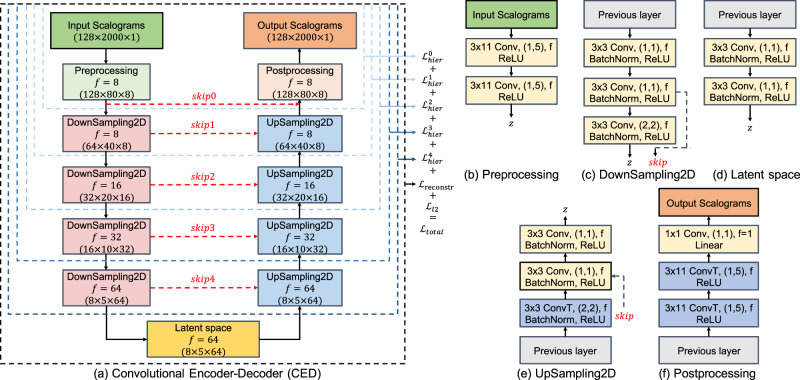
The convolutional encoder-decoder (CED) model for predicting fault friction from scalograms, obtained from kinetic energy (simulation) and acoustic emission (experiment). **a** CED model architecture. The encoder branch contains a **b** Preprocessing block and **c** four DownSampling 2D blocks that populate the **d** latent space. The decoder branch reverses the procedure using **e** four UpSampling 2D blocks and a **f** Postprocessing block. The encoder and decoder models are connected by skip connections (red dashed lines) between the downsampling and upsampling blocks as shown in **a**. The number of filters (*f*) for each block are shown in **a**. The image size after each layer block are provided in parentheses. The blue dashed lines indicate the sub-models used when computing the hierarchical components^39^ and the associated training loss function to obtain the total loss (*L*_total_). The model layer notations are Conv (convolutional layer), ConvT (convolutional transpose layer), BatchNorm (batch normalization layer), linear (linear connected layer), and ReLU (rectified linear unit).

**Fig. 4:**
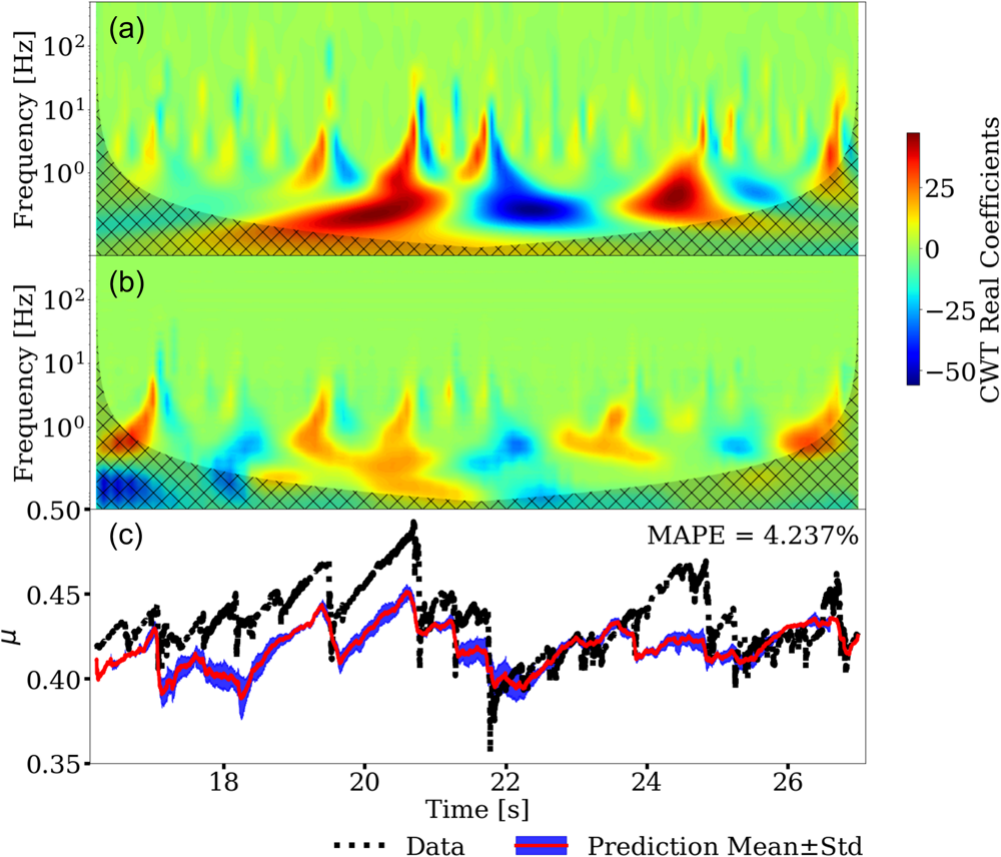
Instantaneous frictional coefficient prediction from the convolutional encoder-decoder (CED) model trained on Finite-discrete element method (FDEM) simulation data. The **a** input and **b** predicted scalograms are shown, with the color bar indicating the continuous wavelet transform (CWT) real coefficients. The cross-hatched region in **b** indicates the cone of influence where edge effects are important. The predictions from the CED are made applying sliding windows with 2 s length and step size of 0.2 s. The predicted scalogram is the average of all sliding windows. **c** The numerical simulation data (black line) and model-predicted friction coefficient *μ* from the inverse of the scalogram is shown in red with the blue region indicating 1-standard deviation for the predictions in the overlapping windows. The mean absolute percentage error (MAPE) is listed for the numerical simulation and predicted values.

Data from the numerical simulation and laboratory experiment (Fig. 2) are used by the CED model (Fig. 3) that is fully described in the Methods section. The supervised learning approach is a regression procedure, using the AE from experiment or the kinetic energy from simulation to predict the instantaneous characteristics of slip, specifically the coefficient of friction. As a point of reference for the transfer learning approach, the results shown in Fig. 4 are produced by training, validating, and testing entirely on the numerical simulation data. The predicted friction coefficient captures the general slip trends including many frictional failures. However, the prediction results are modest as reported by the mean absolute percentage error (MAPE) of 4.237% for the numerical simulation data.

**Fig. 5:**
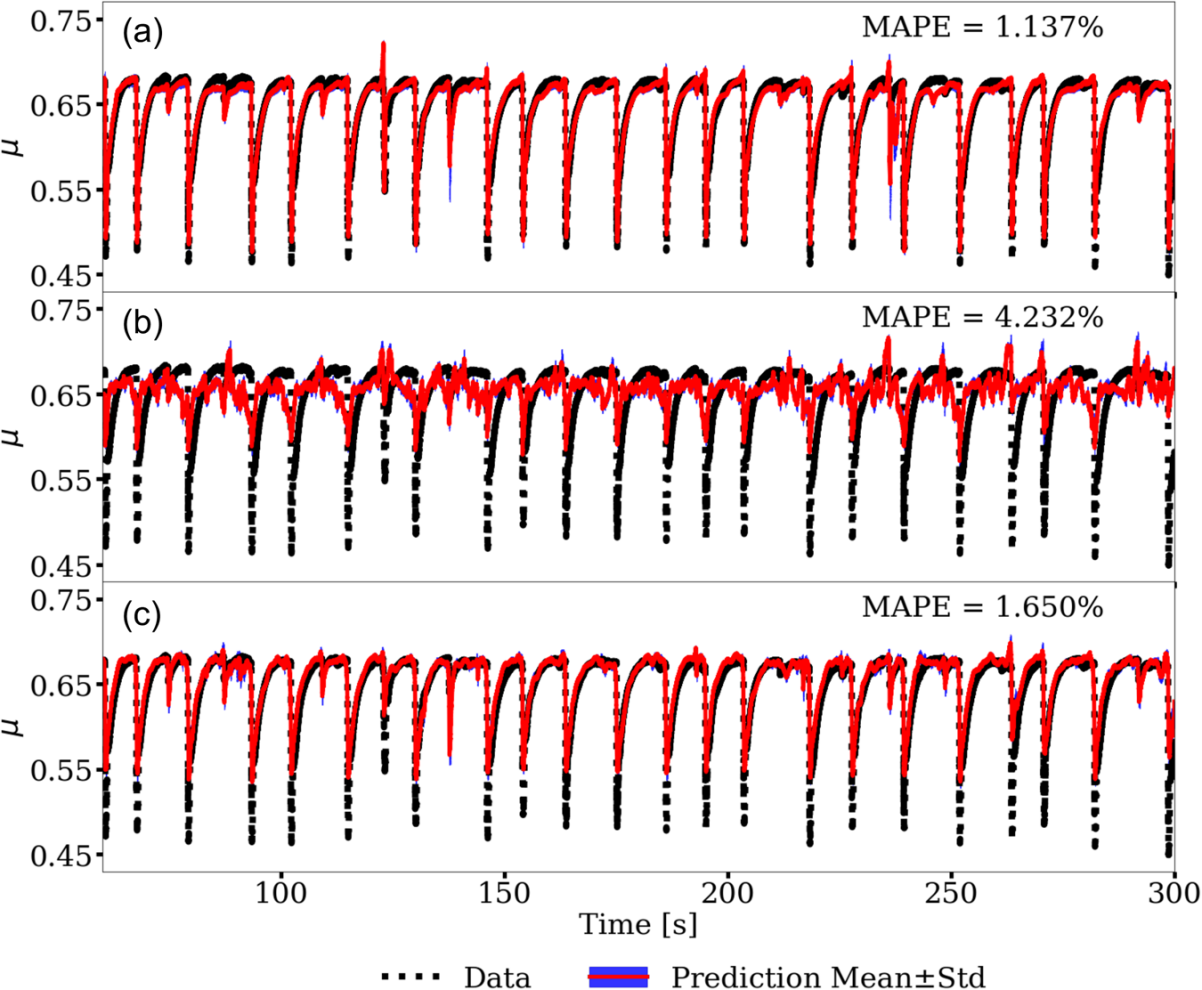
Evolving friction time series predictions from convolutional encoder–decoder (CED) models. **a** Model trained and tested on the laboratory data. **b** Model trained on simulations and tested on laboratory data. The first 20% of the acoustic emission (AE) signal (0–60 s) was used for training to construct the model. **c** Cross-trained model. Model trained on the simulations, then fixing the encoder and decoder layers, with the model then additionally trained on the bottleneck (latent space) applying a portion of the laboratory (experiment p4677) data. The mean absolute percentage error (MAPE) is listed for each prediction. See “Methods” section for details.

As a second point of reference, the same procedure is followed using only the laboratory AE and friction data to train a separate CED model. The first 20% of the AE signal (0–60 s) is used for training data. The friction predictions from the testing data produce a MAPE of 1.137% (Fig. 5a). The model performs very well for estimating the variations in friction coefficients and capturing the frictional failures associated with slip events.

For the first transfer learning exercise, we use the model trained on simulation data and apply it to predict the friction in the laboratory experiment—the trained model uses the experiment AE as input and the label is the friction from experiment. We emphasize that the CED model never sees experimental data during training with the simulation data. The predictions show a decrease in performance with a MAPE of 4.232% (Fig. 5b) when compared to the model trained solely on the laboratory data. The maximum friction drop, which has an equivalence to event moment (moment = GAu; where *G* is the gouge shear modulus, *A* is slip area and *u* is the fault displacement), is consistently less than that measured from the experiment. Under-prediction of the event moment is a common problem with many ML models when applied to the bi-axial shear data^8,13^, without considering transfer learning. Nonetheless, we find the timing and scale of the predictions are surprisingly good considering the significant differences between the FDEM numerical simulation and the laboratory shear experiment.

With an eye to faults in Earth where obtaining sufficient training data is a challenge, we introduce transfer learning by cross-training the model. Here, we allow the latent space of the CED to be trained on limited laboratory data, while fixing the encoder and decoder layers that are trained using only the simulation. This approach is an extension of an established transfer learning technique used in image classification tasks, see e.g.,^24,25^. As applied to image classification, the convolutional layers of a model are pre-trained on a large database, e.g., ImageNet^26,27^, and then specific convolutional layers are extracted and held constant, then merged with an additional fully-connected classification layer that is trained with data specific to the problem of interest.

Here, we apply a transfer learning approach in this same spirit. We emphasize that alternatively here, though analogously, it is the encoder and decoder layers that are directly extracted, and only the latent space weights are updated. For the ML models previously trained on the numerical simulation data, all the parameters in the encoder and decoder are rendered non-trainable, while the parameters in the latent space are updated and fit to the training data from laboratory experiments.

The resulting predictions shown in Fig. 5c and are very good with MAPE = 1.650%, which is a significant improvement from the MAPE of 4.232% before cross-training. The model predictions are now comparable to the MAPE of 1.137% obtained when training directly on p4677 laboratory data.

**Fig. 6:**
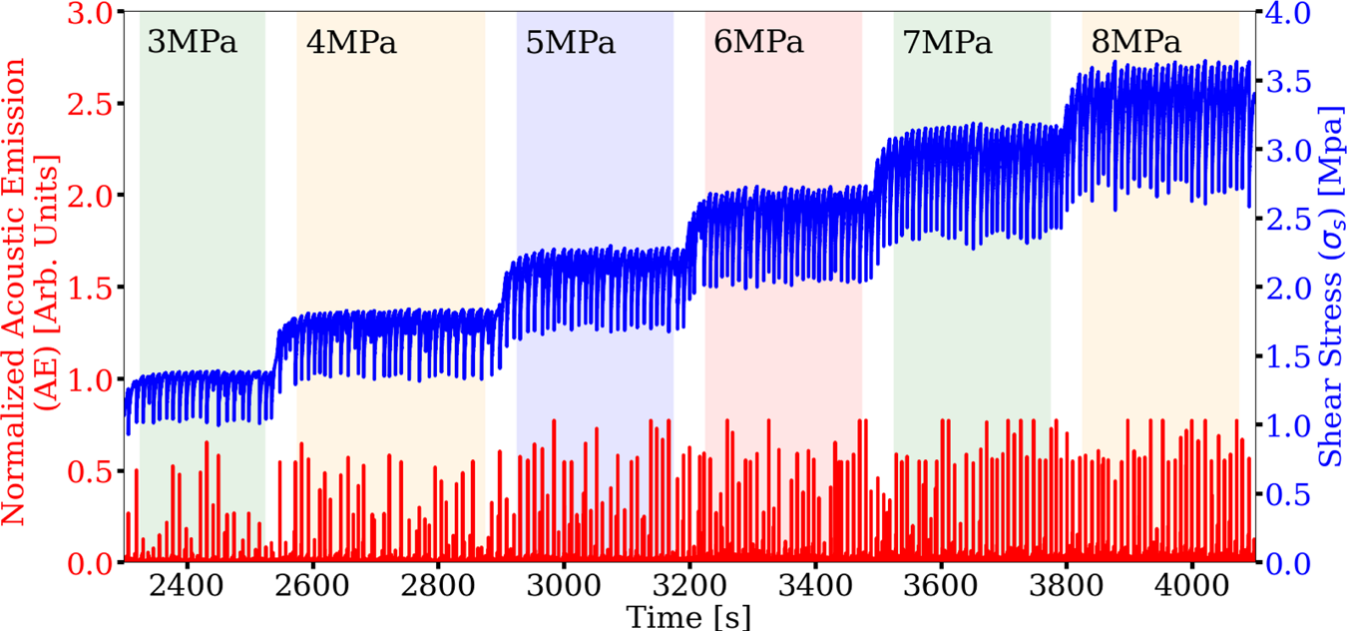
Convolutional encoder-decoder (CED) model generalization. The cross-trained model is rigorously validated using a second laboratory experiment (p4581) as an independent data set. The input signal is the acoustic emission (AE in red, left axis) and the target signal is the friction derived from the shear stress (*σ*_s_ in blue, right axis) at progressively increasing applied normal loads (3–8 MPa), shown in sequence and delineated by different shading.

As a more rigorous test on how well the cross-trained CED model predicts the laboratory experiments, we apply the identical model to a different laboratory experiment conducted in the bi-axial apparatus. These experimental data are never seen during training of the latent space. This second experiment was conducted over a range of confining loads (normal stress) from 3 to 8 MPa (Fig. 6). The only information applied from the different confining load levels are the mean and standard deviation statistics used to normalize the AE and *μ* signals when producing the input scalograms to the model and when reconstructing the output scalograms (see “Methods” section).

**Fig. 7:**
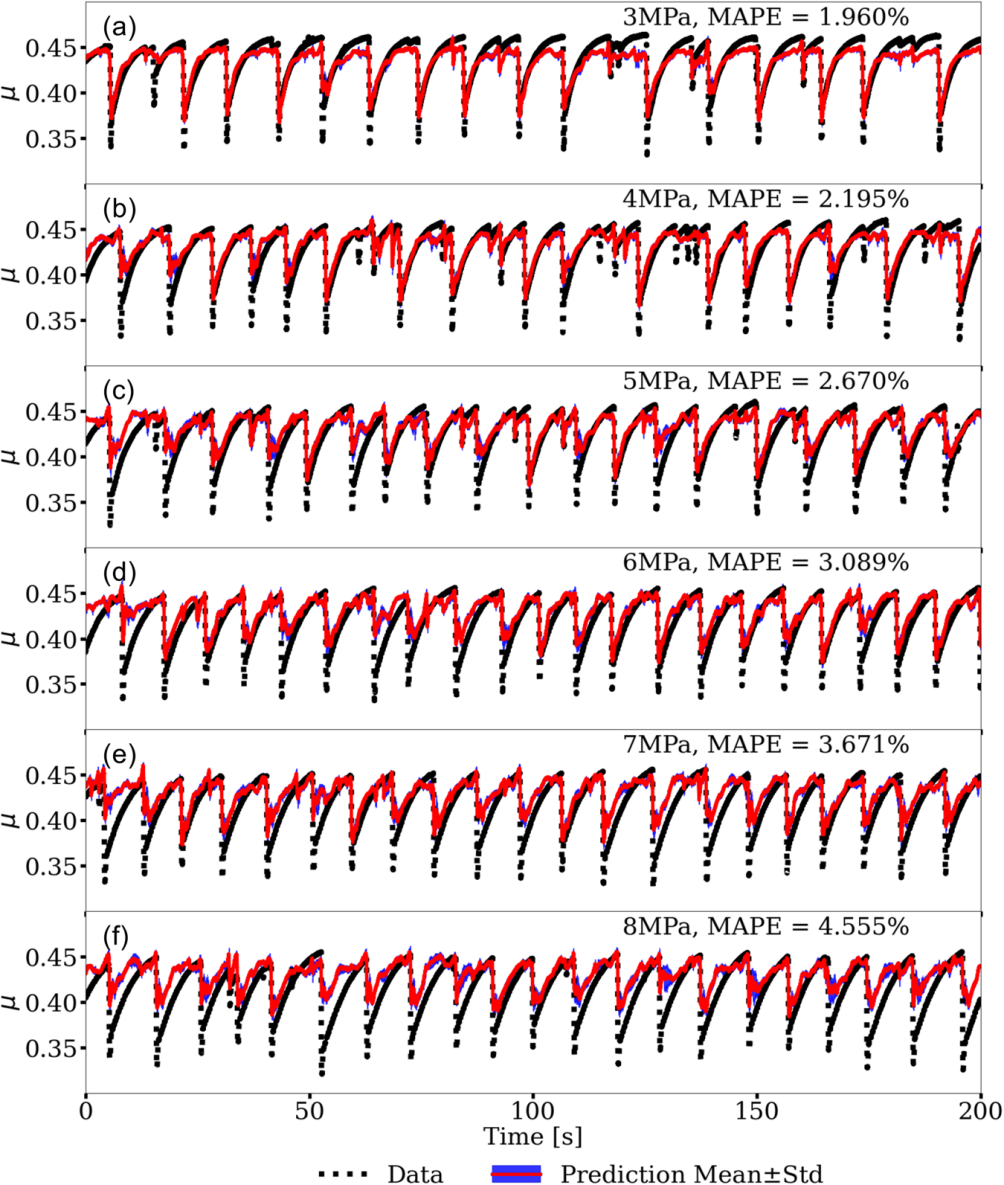
Transfer learning applied to an independent experiment. Shown are predictions from the cross-trained convolutional encoder-decoder (CED) model (experiment p4581) with normal loads that progressively increase from 3 to 8 MPa (see Fig. 6, (a-f), respectively). Each load level is predicted independently using the cross-trained model from simulation (the encoder and decoder) and data from experiment (p4677) conducted at 2.5 MPa. The predictions as manifest by the mean absolute percentage error (MAPE) progressively decrease with increasing load level. Nevertheless, the results show that the transfer learning approach with cross-training of the latent space, which accounts for only 20% of the total CED model parameters, is a powerful approach to predicting the frictional state of the experimental fault.

The predictions applying the cross-trained model to the second experiment are shown in (Fig. 7). The results are remarkably good as indicated by the MAPE’s. The 3 MPa data exhibits the best MAPE, presumably because the confining load is close to the 2.5 MPa stress in the p4677 experiment that was used to train the latent space (Fig. 7a). The model predictions as manifest by the MAPE increase with increasing normal loads. The prediction errors appear to be due primarily to the poor predictions of the frictional failure magnitudes. Nonetheless, the instantaneous slip-event times are captured at all load levels, as are the stress buildups during inter-event periods.

### Transfer learning with extremely limited data in laboratory experiments

**Fig. 8:**
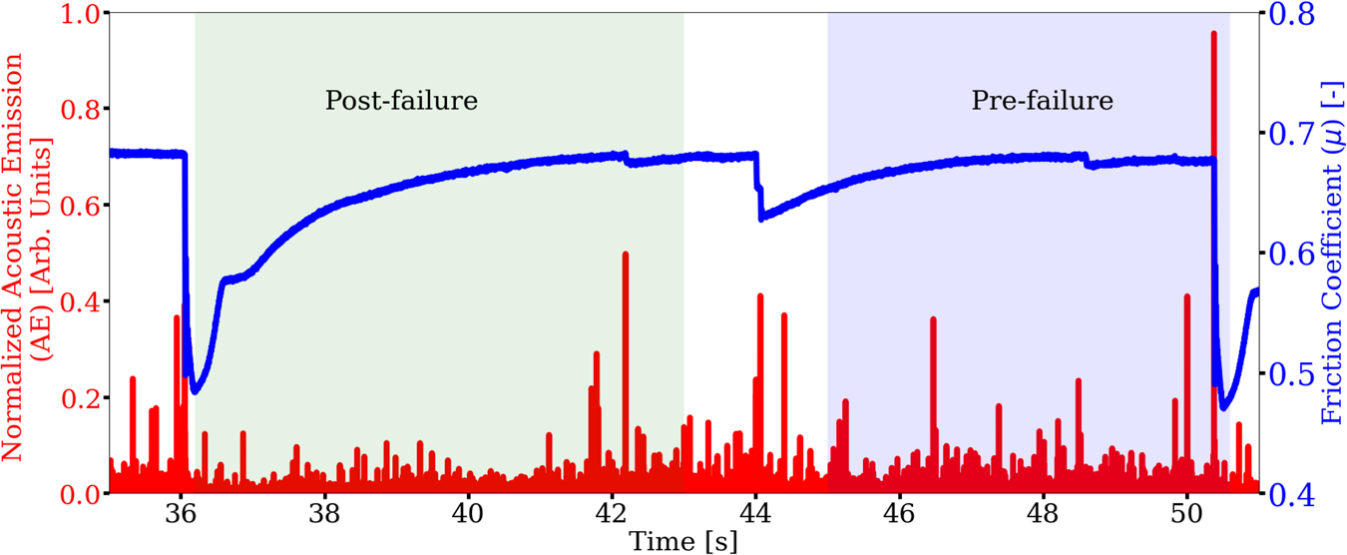
Testing the limits of the transfer learning: convolutional encoder-decoder (CED) model with cross-training of the latent space applying limited portions of acoustic emission (AE) data from a single slip cycle. The model input signal is acoustic emission (AE in red, left axis), and the output signal is the friction coefficient (*μ* in blue, right axis). The portion of the time series shaded light green shows the post-failure data used for the transfer learning exercise and shaded light blue shows the pre-failure data.

Because slip cycles in Earth are so long (decades to 100’s of years) and we rarely have more than a portion of associated seismicity within a full seismic cycle, we conduct a cross training exercise that mimics this data-poor circumstance. We do so by using only limited portions of a single slip cycle from the laboratory experiment for training the model latent space. Specifically, we train the latent space by applying only the post-failure or the pre-failure *μ* signals from experiment p4677 data (Fig. 8). The post-failure comprises the time-period when the shear stress is increasing relatively rapidly following the previous slip event. The pre-failure period comprises the period when the fault is late in the cycle, near-critical state, and beginning to nucleate^21^. The model encoder and decoder trained with the simulation data again remain unchanged. The model is trained and validated using 90% and 10% of the data, respectively, for the pre-failure and post-failure analysis. Because the available data only spans a short time interval, the size of the sliding windows is reduced from 2 to 0.4 s and the step size is reduced from 0.2 to 0.1 s (the sizes have no significant impact on the model performances, see Methods). To prevent over-fitting, the training is terminated when the validation does not reduce for 100 epochs.

**Fig. 9:**
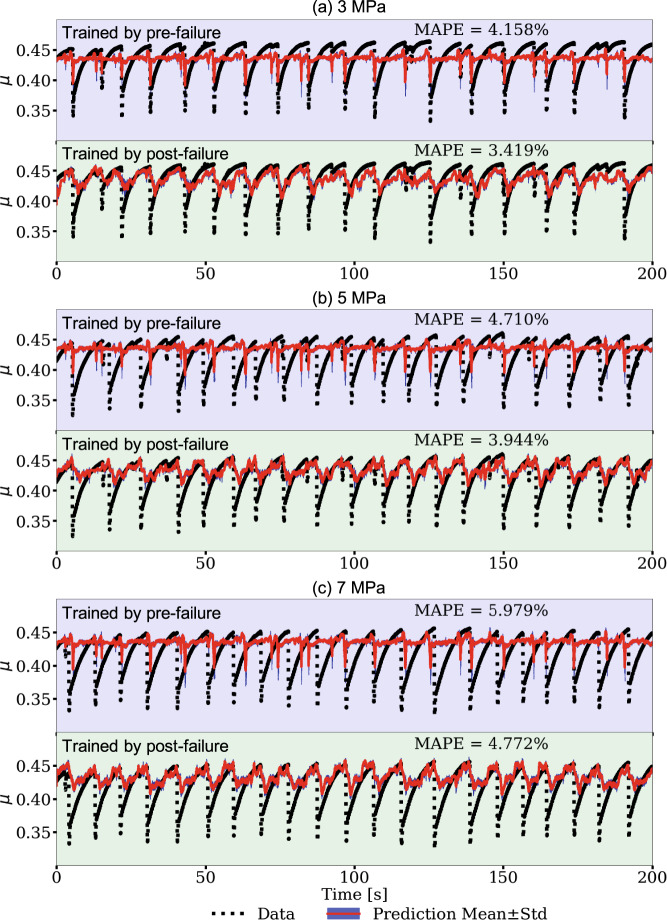
Model cross-training applying limited portions of the experimental slip cycle. Two models are trained and in both cases only the latent space is trained using data from experiment p4677 applying the following criteria. One model is trained applying acoustic emission (AE) data from the post-failure portion of the slip cycle, comprising the time-period when the shear stress is increasing relatively rapidly. The second model is trained applying AE data from the pre-failure period comprising the period the fault is in a critical state and nucleating. The model encoder and decoder trained applying the simulation data remain unchanged. Friction predictions on data from the experiment p4581 testing data set for pre-failure at applied loads of 3, 5, and 7 MPa are shown in each top row of (**a**–**c**) shown in light blue and post-failure in each bottom row of (a, b, c) shown in light green. The shading of the panels correspond to the portion of training data highlighted in Fig. 8. The mean absolute percentage error (MAPE) is shown for each respective load level and model.

After cross-training the latent space using the two data sets from experiment p4677 (post-failure and pre-failure) to produce two separate CED models, the models are used to predict the friction in the second experiment, p4581, for 3, 5, and 7 MPa applied load levels, on the post-failure and pre-failure signals. The results are shown in Fig. 9. As before, the magnitude of the frictional failures are not well predicted—otherwise the trained models perform surprisingly well in both cases. The procedure is repeated using the laboratory data, without transfer learning, to show the improvements in the forward predictions when data is extremely limited (Fig. S1). The results applying the post-failure data are slightly better than that from the pre-failure training data, suggesting the model has learned more frictional states during latent-space training. As anticipated, the model using experiment p4677 and trained applying six full cycles provides the most robust result; however, the model results with extremely limited training cross-training data are very encouraging.

### Transfer learning predicting time to failure in laboratory experiments

**Fig. 10:**
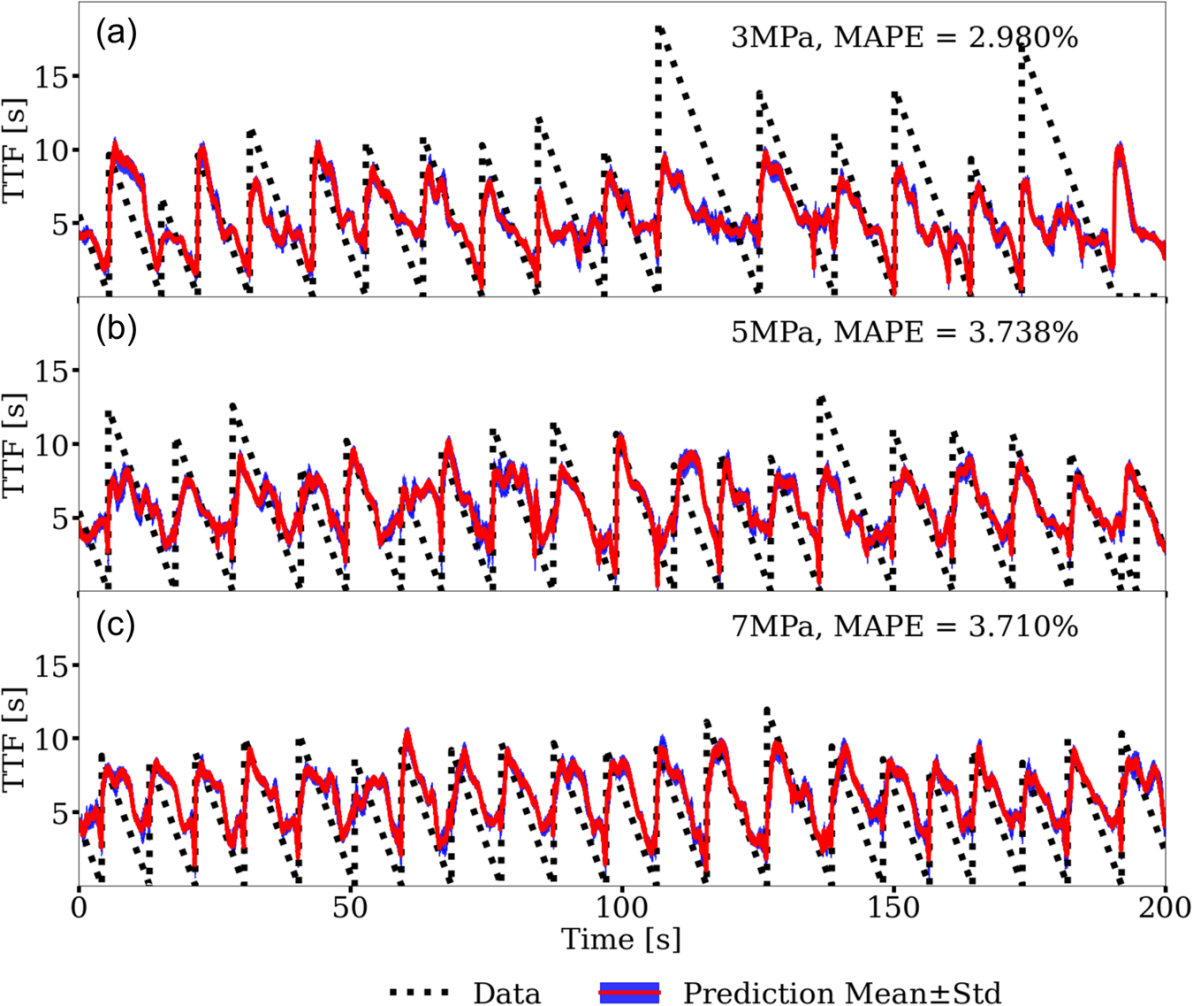
Predictions of time-to-failure (TTF) applying the transfer learning, cross-trained model. Predictions of TTF for laboratory p4581 data at **a** 3 MPa, **b** 5 MPa, and **c** 7 MPa confining loads using transfer learning of the convolutional encoder-decoder (CED) model trained on finite-discrete element method (FDEM) data. Only the latent space is trained on laboratory p4677 data for the TTF predictions. The black dashed line shows the ground truth TTF derived from the experiment. The red curve shows the model predicted TTF with 1-standard deviation shown in blue. The mean absolute percentage error (MAPE) is shown for each load level.

Transfer learning can also be applied to predicting other output time series (see e.g.,^4,6^). Here we showcase the predictions on the signals of time-to-failure (TTF). Failure times are defined as when the time derivative of the *μ* signal is below −10/*s*. The raw AE signal is used as input, just as for the instantaneous predictions of the friction coefficient. The encoder and decoder models are again directly applied from the CED model trained on the numerical simulation data for the task of predicting the *μ* signal. Next, the latent space is trained to fit the TTF training data from experiment p4677 (the first 20% of the signal, from 0 to 60 s, including six stick-slip cycles). Data from p4581 are again used for testing purposes only. The predictions are illustrated in Fig. 10 for 3, 5, and 7 MPa load levels. The predictions are good, if not perfect considering the task, as underscored by their respective MAPE scores. Indeed they are notable considering the they are obtained from cross-training and transfer learning. Repeating this procedure using only laboratory data, without transfer learning, indicates the model can better estimate the full cycle with values extending to zero seconds (Fig. S2) and agrees with initial studies extracting information from the continuous waveforms^5^.

## Discussion

The predictions of the instantaneous friction obtained applying transfer learning from FDEM simulations to laboratory data from the bi-axial shear device are surprisingly good. When model cross-training of the latent space is then applied, the predictions improve significantly. Further, when we apply the same cross-trained model to the second experiment conducted at multiple applied loads, the model predictions are still surprisingly good—there exists a larger misfit with increasing load, but the timing of the event is accurate regardless of the under-prediction in friction failure magnitude. The results are even more remarkable considering the FDEM simulation was not meant to directly simulate the experiment—material properties and dimensions of the fault gouge and shear-blocks were considerably different. Indeed, the results indicate the simulations contain an AE (kinectic energy) evolution captured in the spectral characteristics that can predict the actual AE in experiment. The results suggest the simulations, despite the differences, provide a sufficient distribution of behaviors for the models to learn and reproduce the laboratory behavior—the FDEM simulation exhibits more complex slip behaviors than the experiments in regards to the range of interevent times. Consequently, the trained model is able to predict the simpler behavior with more quasi-periodic interevent times exhibited by the experimental data. The slip frictional-failure magnitude predictions are less accurate than the timing—the full range of frictional failures observed during sliding is under-predicted. Knowing this, one could conduct simulations that produce larger frictional failures to determine if this improves the laboratory failure predictions. It is also interesting that the latent space trained on the post-failure laboratory data produces better predictions than pre-failure training. This may give us clues in Earth regarding where we might anticipate better predictions.

The transfer learning and cross-training results are encouraging. As previously noted, for real-world seismic applications stick-slip repeat cycles can be on timescales ranging from several decades to centuries. Thus in general, available geophysical recordings only include a partial earthquake cycle. Based on the work presented here, we imagine the following as one potential scenario for addressing the sparse data problem. After selecting a fault in Earth to be characterized, numerical simulations of numerous earthquake cycles will be conducted. A deep learning model is then developed applying simulation results, where continuous AE data are used as model input and fault displacement is used as target. Once the model is trained applying simulation results, we cross-train the model latent space with continuous seismic data recorded from the actual fault. This model can then be tested using continuous seismic data not used during the training (e.g., a different time period), to determine if the model can predict geodetic measured surface displacement for that time period. This is one potential approach however there exist parallel approaches one could imagine and test as well. The general transfer learning and cross-training approach may be of great value as we address evolving fault slip and earthquake hazards in the real Earth.

## Methods

### Numerical simulation and laboratory experiments

Numerical simulations of a laboratory experiment performed by our group (Gao et al.^22^) were obtained by applying the combined finite-discrete element method (FDEM) using the Hybrid Optimization Software Suite package (HOSS)^28^ (Fig. 1). The FDEM used in this study was originally developed to simulate continuum to discontinuum transitional material behavior^29^. FDEM combines the algorithmic advantages of the discrete element method with those of the finite element method. In FDEM, each discrete element is comprised of a subset of finite elements that are allowed to deform according to the applied load, which is particularly useful in capturing deformations in the fault gouge material as well as at the gouge particle–plate boundary.

The FDEM model was applied to simulate a two-dimensional, photoelastic shear laboratory experiment conducted by Geller and others^23^. Two-dimensional plane stress conditions were assumed and the model comprised 2817 circular particles confined between two identical plates. Three thousand bi-dimensional particles with diameters of 1.2 and 1.6 mm were used, respectively (1500 of each). The plates had dimensions of 570 × 250 mm. At the plate interfaces semi-circular shaped “teeth” were placed to increase friction between plates. The particles had Young’s modulus and Poisson’s coefficient of 0.4 GPa and 0.4, respectively, while the plates had Young’s modulus and Poisson’s coefficient of 2.5 MPa and 0.49, respectively, far smaller than those used in the bi-axial shear experiment described below. Shearing velocity was 0.5 mm/s.

The laboratory data^21,30–33^ were obtained applying a bi-axial shear apparatus (Fig. 1). Laboratory experiments fail in quasi-periodic cycles of stick and slip that mimic to some degree the seismic cycle of loading and failure on tectonic faults. The apparatus comprises a central steel block that is driven at fixed loading velocity of 10 μm/s for the experiment. This loading imparts shear stresses within two gouge layers that are 100 mm square with an initial thickness of 5 mm. The gouge layers are located on either side of the central driving block and confined by a second steel layer of 20 mm thickness. The gouge consists of monodisperse glass beads of 104–149 μm diameter with Young’s modulus of 70 GPa and Poisson coefficient of 0.3; the steel blocks have Young’s modulus of approximately 180 GPa and Poisson’s coefficient of approximately 0.29. A load-feedback servo control system maintains a fixed normal stress of 2.5 MPa for experiment p4677, while measuring shear stress throughout the experiment. For experiment p4581, progressively larger loads were applied, and at each load level, steady state was achieved before a change to the successive load level. The shearing speed was 5 mm/s for both experiments. Mechanical data measured on the apparatus throughout the experiments included the shearing block velocity, the applied load, the gouge layer thickness, the shear stress, and the coefficient of friction. Continuous AE emissions from the fault zone seismic wave radiation were recorded with piezo-ceramics embedded inside blocks of the shear assembly^34^.

We note that the AEs from the FDEM simulations were not propagated as elastic waves in the model. We assume the kinetic energy obtained from the fault simulations as being equivalent to the AEs recorded on the experimental shear apparatus based on previous analysis^22^. Used here as an equivalent quantity to the AE is the kinetic energy (*E*_k_) summed from the entire system. Since the plates and particles work together as an ensemble, it is the aggregate energy evolution that governs the stick-slip behavior in granular fault gouge. In the bi-axial experiment, the source of the AE signal is at the grain contact level^35^. Fault gouge contacts broadcast AE independently and/or simultaneously^35^, and displace the sideblocks equivalently along the dimensions of the block due to the extreme stiffness of the steel, in analogy to the *E*_k_ behavior in the simulation. Thus, the *E*_k_ is approximately equivalent to the magnitude of the continuous AE time series (norm of the acoustic emission recorded by the two channels of the lab experiments), which is the source of elastic waves. We say approximately because there is a modest amount of wave dissipation and wave scattering during wave propagation in the experiment from the fault gouge layer through the steel plates to the detectors.

The density distributions of the normalized inputs and outputs for the two data sets vary the most in the output friction values (Fig. S3). The input data show quite similar distributions, suggesting the model can extract the needed features from the simulation to make a prediction on the experiment. The friction values show much wider distribution in the simulation data compared to an apparent concentration around a peak from the laboratory data. The two-sample Kolmogorov–Smirnov (KS) test shows that the distributions of the FDEM and Lab friction data are statistically different (KS statistic = 0.258 and *p*-value = 0.0). This inconsistency helps validate our choice to use the transfer learning for adjusting the weights in the latent space to account for this out-of-distribution of lab data from simulation data.

### Training, validation, and testing data

The continuous time series signals (AE, kinetic energy, and friction coefficient) from the experiment and FDEM simulation are converted into scalograms using the Continuous Wavelet Transform (CWT; see ref. ^36^ for a comprehensive description of the method) to utilize the time-frequency signal strength in the CED models. We adopt the real Ricker (Mexican-hat, DOG (*m* = 2)) wavelet for the CWT, which is commonly used in analyzing seismic data^37^. For comparison we also tested the Morlet wavelet and found the Ricker to produce improved MAPE results. The reconstruction of the signal from the scalograms (inverse CWT) is the sum of the real part of the wavelet transform over all scales.

For the FDEM simulation, the CWT is performed on the training/validation/testing (60/20/20% split) segments of the kinetic energy (*E*_k_) and friction (*μ*) time series. Scalograms are calculated using moving windows with a size of 2 s and step of 0.2 s. The sliding window size does not impact the accuracy of the CED model, see “Methods” section Testing model design and training procedure. For a sampling frequency of *f*_s_ = 1000 Hz, each scalogram is 128 × 2000. The procedure produces 73 and 19 pairs of input *E*_k_ and output *μ* scalograms for the training and validation, respectively.

The training data is augmented by producing additional noisy *E*_k_ input signals. The procedure is as follows: (1) take the Fast Fourier Transform (FFT) of the original signal data, (2) shuffle the positive-frequency terms of the imaginary coefficients, (3) set the negative-frequency terms to the opposite of the shuffled terms, (4) and perform the inverse FFT to produce a new *E*_k_ signal with the same amplitude spectrum and random phase. The procedure is repeated three times for the training signal and the final training data contains 292 scalogram pairs.

The CWT transform procedure is applied to the laboratory experiment p4677 acoustic emission (AE) and friction (*μ*) time series to produce training/validation/testing (20/20/60% split) data. The scalogram dimensions are the same as the numerical simulations. The final data set contains 292 pairs of input AE_norm_ and output *μ* scalograms for the training and validation data. Scalograms are calculated for the laboratory experiment p4581 and only used as testing data for experiments conducted at different normal stresses.

Before applying the CWT, all input and output signals are normalized by subtracting the mean and dividing by the standard deviation using the statistics extracted from the training signal data. For FDEM data, the statistics are 3.28E−4 ± 5.00E−4 for the input *E*_k_ signals and 4.23E−1 ± 2.52E−2 for the output *μ* signals. For transfer learning on the p4677 data, the statistics from the training signals (0–60 s, including six stick-slip cycles) are 8.932 ± 14.900 for the input AE signals and 0.657 ± 0.0382 for the output *μ*. In the cases of limited sub-cycle data, the post-failure training signal has statistics of 7.712 ± 10.667 for AE and 0.641 ± 0.0440 for *μ*, and the pre-failure training signal has statistics of 10.205 ± 20.137 for AE and 0.667 ± 0.0377 for *μ*. When making predictions using the laboratory p4581 data with increasing normal loads, the statistics are extracted from the first 20% of the 3MPa signal (from 0 to 40 s, including five stick-slip cycles) to obtain 17.776 ± 46.700 for AE and 0.433 ± 0.0230 for *μ*. For the TTF statistics the values are 4.816 ± 3.257 on the p4677 data and 4.817 ± 2.873 on the p4581 data, extracted from the same aforementioned signal segments.

### Convolutional encoder-decoder model and transfer learning

The CED architecture is composed of an encoder branch containing the salient features that feed to a latent space, and a decoder branch to construct the output variable. The input signal is passed to an encoding branch with a preprocessing block containing two convolutional layers and a rectified linear unit (ReLU) activation function (Fig. 3). Preprocessing is used to reduce the image size in the time dimension by a factor of 25. This is passed through four downsampling blocks containing three convolutional layers, each with batch normalization, ReLU activation, and a skip connection. The latent space contains two convolutions and a ReLU activation. The decoding branch reverses the encoding using convolutional transpose layers. The postprocessing contains two convolutional transpose layers to obtain the original dimensions. The dimension of each layer, i.e., the filter size, depth, and skip connections are labeled in Fig. 3. The model contains five “skip” connections^38^ that directly link the weights from the downsample blocks in the encoder to the upsample blocks in the decoder at each level. The trainable weights are initialized using glorot_uniform and the biases are nontrainable and set to zero. The CED model contains 363,696 trainable parameters, with 73,984 in the latent space.

The model design utilizing the CWT images with skip-connections yields improved prediction accuracy over simpler more-standard approaches, such as alternatively directly inputting the waveform data and using 1D convolutions. As a point of comparison, a convolutional encoder model followed by a set of fully connected layers has 1–2 orders of magnitude more trainable parameters and does not outperform the adopted design. The number of filters and layers of the CED model (see Fig. 3) have been reduced and the performance was compared to validate the final model selection (Table S5). The adopted design produces the best overall performance.

Loss functions are calculated hierarchically for each pair of encoder/decoder blocks. This type of hierarchical regularization was recently introduced by Wang et al.^39^ to provide better interpretability and generalizability of CED models for learning fluid-flow patterns in complex rock pore-structures. This regularization is found to improve the MAPE accuracy in predicting FDEM test data by 1% and provides a similar level of MAPE for the transfer learning. The total loss is calculated as ${L}_{{{\mbox{total}}}}={L}_{{{\mbox{hier}}}}^{0}+{L}_{{{\mbox{hier}}}}^{1}+{L}_{{{\mbox{hier}}}}^{2}+{L}_{{{\mbox{hier}}}}^{3}+{L}_{{{\mbox{hier}}}}^{4}+{L}_{{{\mbox{reconstr}}}}+{L}_{{{\mbox{l2}}}}$. Where ${L}_{\,{{\mbox{hier}}}\,}^{i}$ is the mean square error (MSE) between the target and predicted values for each sub-model linked with a “skip” connection (Fig. 3a). *L*_reconstr_ is the reconstruction loss using the entire CED model. And *L*_l2_ is the loss associated to the L2 regularization using a penalty multiplier^40^ set to 1E−5. After the initial training, the “skip” connections are deactivated so that information only passes down the encoder, through the latent space, and up the decoder for a prediction.

The model is trained with a NVIDIA Tesla P100 GPU using 292 pairs of scalograms with a batch size of 8, the Adam optimizer, and a learning rate of 1E−3. Validation is performed with 19 pairs of scalograms. The training is terminated when the reconstruction loss on the training data is below 0.1 and the validation reconstruction loss does not diminish for 100 epochs. The model with the lowest validation loss is used as the final CED model. Transfer learning is applied using the laboratory p4677 data. A new CED model is created with the weights from the final model trained on the FDEM simulation data. All trainable weights, except the latent space, are rendered non-trainable and held constant while the latent space is further trained with the laboratory data. Since the encoder and decoder branches are non-trainable layers, the total loss is *L*_total_ = *L*_reconstr_ + *L*_l2_ and the early stopping is the same. The initial training on the FDEM simulation data takes approximately 15 min and the latent space cross-training on the laboratory data takes about 10 min for full convergence.

### Testing model design and training procedure

Due to random variable initialization and the stochastic nature of training a neural network, repeating the training procedure gives different results and variations in the overall performance. We performed multiple runs of the same transfer learning workflow to assess the average performance of the trained CED models to assess these expected variations. (1) Five runs are performed starting from the same initialized model weights and no noisy data augmentation is added (Table S1). (2) Five runs are performed starting from the same initialized model weights and including the noisy data augmentation (Table S2). (3) Ten runs are performed starting randomly initialized model weights and including the noisy data augmentation (Table S3). The process is then repeated using laboratory data obtained at different confining stress (Fig. S4). The results of these tests show the random initialization and shuffling of the batches produce discrepancies between the model predictions, and increasing the noise through data augmentation reduces the variance and improves accuracy. The main results presented come from the CED model trained in Run No. 8 (Table S3) with an overall accuracy nearest to the mean performance of the ten separate runs with random initial weights and random noisy data augmentation.

The input data length is tested to evaluate the effect of the size of the sliding window on the model predictions. We performed six runs with randomly initialized model weights and noisy data augmentation, using different sizes of the sliding windows 0.4, 0.8, 1, 3, 4, and 5 s (Table S4). These tests indicate the window size produces little variation in the final results shown using a 2 s sliding window (Table S3). The transfer learning approach is robust to the hyperparameter of sliding window size.

## Supplementary information

Supplementary Information

## Data Availability

The numerical FDEM data used in this study are publicly available at 10.5281/zenodo.1248174. The experimental used in this study from experiments p4677 and p4581 are hosted by Chris Marone at the Pennsylvania State University, available at https://sites.psu.edu/chasbolton/.

## Code availability

This study was performed using the Python package NumPy, TensorFlow and PyCWT. The Python code is under restricted access and is not available for public release due to institutional regulations at Los Alamos National Laboratory.
