# Supplementary material for: Predicting fault slip via transfer learning
Source: Nat Commun. 2021 Dec 16;12:7319. doi: 10.1038/s41467-021-27553-5 (PMC8677738; doi:10.1038/s41467-021-27553-5)
Supplement: Supplementary file 1 — Supplementary Information [file 41467_2021_27553_MOESM1_ESM.pdf]

Supplementary Information for Predicting Fault Slip via Transfer Learning

Kun Wang, Christopher W. Johnson, Kane C. Bennett, and Paul A. Johnson *

This Supplementary Information includes Figures S1-S4 and Tables S1-S5.

*Los Alamos National Laboratory, Los Alamos, New Mexico, USA. paj@lanl.gov

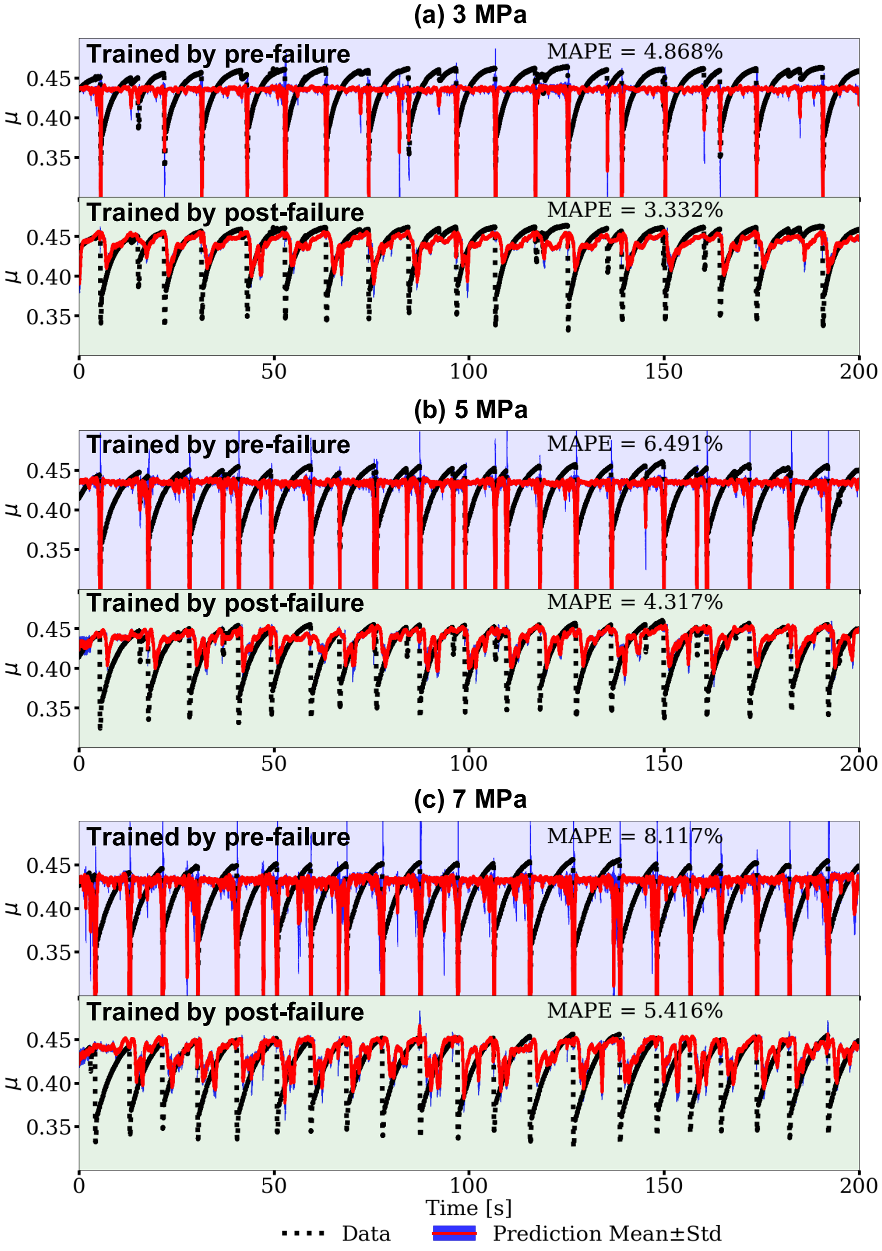

Figure S1: Direct model training applying limited portions of the experimental slip cycle. Friction predictions on data from the experiment p4581 testing data set for pre-failure at applied loads of 3,5, and 7 are shown in each top row of (a, b, c) and post-failure in each bottom row of (a, b, c).

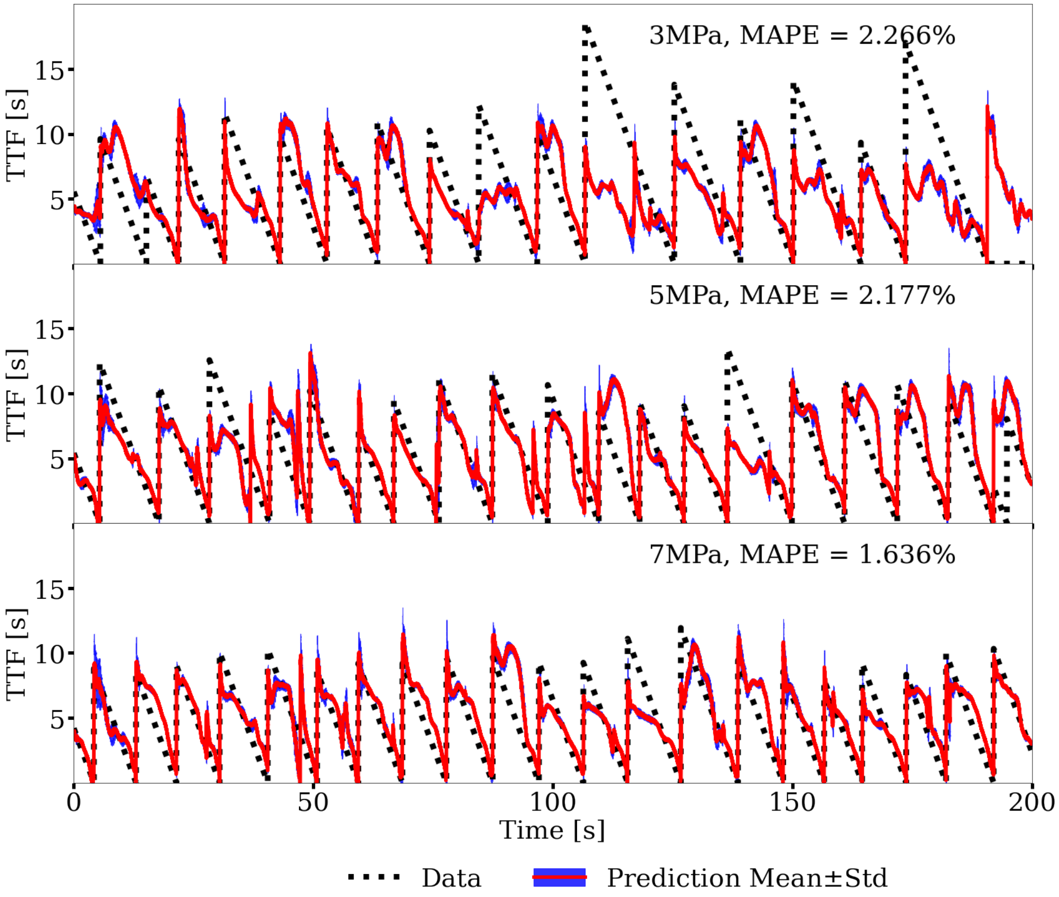

Figure S2: Predictions of time-to-failure (TTF) for laboratory p4581 data at (a) 3 MPa, (b) 5 MPa, and (c) 7 MPa confining loads applying the model directly trained with p4677 TTF data. The blue dashed line shows the ground truth TTF derived from the experiment. The red curve shows the model predicted TTF.

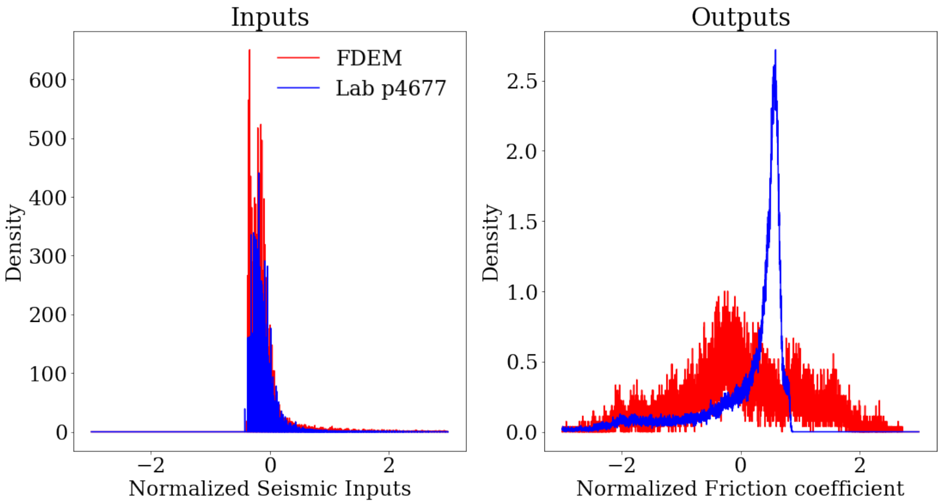

Figure S3: Histograms of the input and output values from the FDEM simulation and Lab p4677 data. The two-sample Kolmogorov-Smirnov (KS) test on the normalized output data shows that the FDEM and Lab distributions are not identical (KS statistic = 0.258 and p-value = 0.0). For the FDEM data, the input kinetic energy signal is normalized by the mean $\overline{KE} = 0.000317$ and the standard deviation $KE_\sigma = 0.000584$, and the output friction coefficient is normalized by $\overline{\mu} = 0.428$ and $\mu_\sigma = 0.0237$. For the Lab p4677 data, the input acoustic emission signal is normalized by $\overline{AE} = 8.838$ and $AE_\sigma = 21.079$, and the output friction coefficient is normalized by $\overline{\mu} = 0.651$ and $\mu_\sigma = 0.0419$.

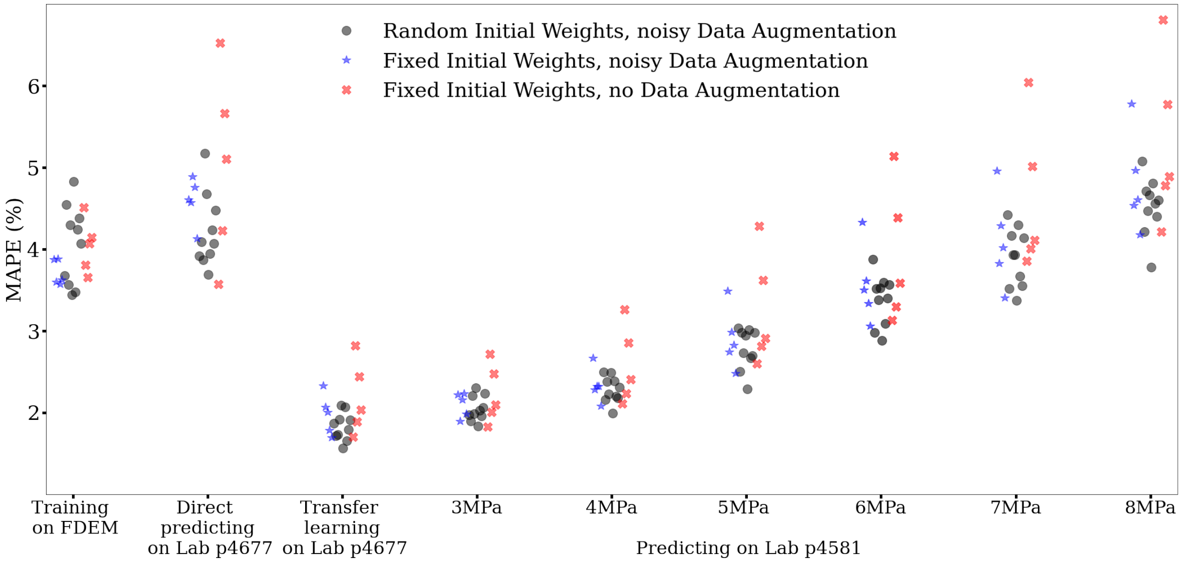

Figure S4: Distribution of MAPE on FDEM, Lab p4677 and Lab p4581 data from models trained in different runs of the proposed transfer Learning workflow. All R^2 and MAPE values are provided in Tables S1, S2, and S3.

	Training on FDEM	Direct predicting on Lab p4677	Transfer learning on Lab p4677	Predicting on Lab p4581					
Run No.	Validation &Testing	Validation &Testing	Validation &Testing	3MPa	4MPa	5MPa	6MPa	7MPa	8MPa
1	-0.649<br>4.508%	0.303<br>3.572%	0.856<br>1.700%	0.826<br>1.828%	0.793<br>2.111%	0.731<br>2.600%	0.653<br>3.132%	0.557<br>3.854%	0.492<br>4.215%
2	-0.0874<br>3.805%	-0.583<br>6.524%	0.508<br>2.822%	0.571<br>2.718%	0.442<br>3.259%	0.192<br>4.284%	0.00691<br>5.140%	-0.198<br>6.041%	-0.449<br>6.806%
3	-0.0578<br>3.656%	0.0815<br>4.228%	0.813<br>1.889%	0.786<br>2.004%	0.766<br>2.235%	0.663<br>2.811%	0.604<br>3.295%	0.483<br>4.004%	0.318<br>4.779%
4	-0.458<br>4.067%	-0.392<br>5.662%	0.625<br>2.442%	0.677<br>2.476%	0.590<br>2.853%	0.397<br>3.616%	0.254<br>4.388%	0.118<br>5.014%	-0.088<br>5.771%
5	-0.387<br>4.143%	-0.060<br>5.100%	0.778<br>2.034%	0.771<br>2.094%	0.699<br>2.403%	0.573<br>2.912%	0.461<br>3.583%	0.391<br>4.110%	0.151<br>4.888%
Mean R^2	-0.328 ± 0.225	-0.130 ± 0.320	0.716 ± 0.130	0.726 ± 0.0917	0.658 ± 0.129	0.511 ± 0.195	0.396 ± 0.239	0.270 ± 0.277	0.0848 ± 0.328
Mean MAPE	4.036% ± 0.294%	5.017% ± 1.040%	2.177% ± 0.404%	2.224% ± 0.325%	2.572% ± 0.425%	3.244% ± 0.622%	3.908% ± 0.752%	4.605% ± 0.824%	5.292% ± 0.906%

Table S1: Starting from the same initialized CED model weights, records of 5 runs without random noisy data augmentation (noDA) of the same transfer learning workflow. The performance of the model trained in each run is measured by the coefficient of determination (R^2, top row in each cell) and the mean absolute percentage error (MAPE, bottom row in each cell, in %).

	Training on FDEM	Direct predicting on Lab p4677	Transfer learning on Lab p4677	Predicting on Lab p4581					
Run No.	Validation &Testing	Validation &Testing	Validation &Testing	3MPa	4MPa	5MPa	6MPa	7MPa	8MPa
1	0.0232<br>3.627%	0.162<br>4.127%	0.870<br>1.696%	0.790<br>1.987%	0.787<br>2.080%	0.733<br>2.484%	0.632<br>3.059%	0.601<br>3.406%	0.434<br>4.181%
2	0.102<br>3.572%	-0.0368<br>4.758%	0.818<br>1.785%	0.742<br>2.232%	0.737<br>2.321%	0.665<br>2.827%	0.596<br>3.335%	0.496<br>4.020%	0.405<br>4.605%
3	-0.129<br>3.882%	-0.0436<br>4.886%	0.770<br>2.008%	0.765<br>2.159%	0.736<br>2.324%	0.636<br>2.983%	0.544<br>3.614%	0.443<br>4.288%	0.286<br>4.967%
4	0.117<br>3.600%	0.0629<br>4.574%	0.802<br>2.068%	0.808<br>1.897%	0.781<br>2.283%	0.717<br>2.741%	0.586<br>3.504%	0.564<br>3.828%	0.399<br>4.537%
5	-0.0284<br>3.877%	0.0472<br>4.603%	0.727<br>2.329%	0.746<br>2.218%	0.669<br>2.664%	0.514<br>3.486%	0.351<br>4.327%	0.257<br>4.957%	0.0325<br>5.781%
Mean R^2	0.0169 ± 0.0901	0.0383 ± 0.0753	0.797 ± 0.0478	0.770 ± 0.0254	0.742 ± 0.0423	0.653 ± 0.0778	0.542 ± 0.0994	0.472 ± 0.121	0.311 ± 0.148
Mean MAPE	3.712% ± 0.138%	4.590% ± 0.257%	1.977% ± 0.223%	2.099% ± 0.133%	2.334% ± 0.188%	2.904% ± 0.333%	3.568% ± 0.423%	4.100% ± 0.516%	4.814% ± 0.544%

Table S2: Starting from the same initialized CED model weights, records of 5 runs with random noisy data augmentation (DA) of the same transfer learning workflow. The performance of the model trained in each run is measured by the coefficient of determination (R^2, top row in each cell) and the mean absolute percentage error (MAPE, bottom row in each cell, in %).

	Training on FDEM	Direct predicting on Lab p4677	Transfer learning on Lab p4677	Predicting on Lab p4581					
Run No.	Validation &Testing	Validation &Testing	Validation &Testing	3MPa	4MPa	5MPa	6MPa	7MPa	8MPa
1	-0.0473<br>3.673%	0.262<br>3.913%	0.821<br>1.865%	0.797<br>1.969%	0.720<br>2.492%	0.633<br>3.032%	0.492<br>3.878%	0.423<br>4.418%	0.267<br>5.078%
2	-0.555<br>4.541%	0.344<br>4.089%	0.843<br>1.718%	0.798<br>1.898%	0.775<br>2.157%	0.732<br>2.502%	0.650<br>2.976%	0.580<br>3.517%	0.447<br>4.216%
3	-0.020<br>3.566%	0.197<br>3.865%	0.821<br>1.728%	0.745<br>2.206%	0.723<br>2.378%	0.635<br>2.977%	0.553<br>3.516%	0.439<br>4.166%	0.314<br>4.706%
4	-0.246<br>4.294%	-0.0621<br>5.170%	0.805<br>1.916%	0.787<br>1.985%	0.744<br>2.226%	0.653<br>2.730%	0.559<br>3.378%	0.459<br>3.929%	0.325<br>4.471%
5	0.144<br>3.438%	-0.0891<br>4.678%	0.731<br>2.091%	0.726<br>2.302%	0.671<br>2.490%	0.553<br>2.946%	0.455<br>3.523%	0.390<br>3.928%	0.235<br>4.658%
6	-0.537<br>4.825%	0.471<br>3.686%	0.876<br>1.567%	0.825<br>1.831%	0.793<br>1.989%	0.758<br>2.288%	0.673<br>2.881%	0.618<br>3.374%	0.552<br>3.777%
7	0.0405<br>3.475%	0.176<br>3.946%	0.793<br>2.070%	0.797<br>2.028%	0.750<br>2.385%	0.646<br>3.010%	0.560<br>3.593%	0.446<br>4.295%	0.338<br>4.805%
8	-0.324<br>4.237%	0.294<br>4.232%	0.846<br>1.650%	0.801<br>1.960%	0.764<br>2.195%	0.661<br>2.670%	0.594<br>3.089%	0.528<br>3.671%	0.324<br>4.555%
9	-0.375<br>4.381%	0.251<br>4.068%	0.835<br>1.791%	0.761<br>2.062%	0.740<br>2.179%	0.642<br>2.693%	0.541<br>3.397%	0.544<br>3.548%	0.375<br>4.399%
10	-0.273<br>4.071%	0.159<br>4.477%	0.789<br>1.909%	0.741<br>2.232%	0.734<br>2.308%	0.617<br>2.978%	0.523<br>3.561%	0.441<br>4.138%	0.331<br>4.596%
Mean R^2	-0.219 ± 0.228	0.200 ± 0.162	0.816 ± 0.0377	0.778 ± 0.0306	0.741 ± 0.0319	0.653 ± 0.0543	0.560 ± 0.0628	0.487 ± 0.0715	0.351 ± 0.0860
Mean MAPE	4.050% ± 0.461%	4.212% ± 0.424%	1.830% ± 0.163%	2.047% ± 0.145%	2.280% ± 0.152%	2.783% ± 0.237%	3.379% ± 0.293%	3.898% ± 0.339%	4.526% ± 0.334%

Table S3: Records of 10 different runs with randomly initialized CED model weights and random noisy data augmentation of the same transfer learning workflow. The performance of the model trained in each run is measured by the coefficient of determination (R^2, top row in each cell) and the mean absolute percentage error (MAPE, bottom row in each cell, in %). The bottom 2 cells are the mean and standard deviation.

	Training on FDEM	Direct predicting on Lab p4677	Transfer learning on Lab p4677	Predicting on Lab p4581					
Size of sliding windows	Validation &Testing	Validation &Testing	Validation &Testing	3MPa	4MPa	5MPa	6MPa	7MPa	8MPa
0.4s	-0.610<br>4.801%	0.208<br>4.345%	0.797<br>2.069%	0.724<br>2.278%	0.731<br>2.327%	0.700<br>2.681%	0.581<br>3.413%	0.546<br>3.740%	0.381<br>4.496%
0.8s	-0.478<br>4.688%	0.359<br>3.851%	0.891<br>1.570%	0.819<br>1.882%	0.817<br>1.986%	0.771<br>2.357%	0.658<br>3.103%	0.625<br>3.510%	0.400<br>4.499%
1s	-0.324<br>4.012%	0.0142<br>4.586%	0.837<br>1.802%	0.733<br>2.200%	0.705<br>2.367%	0.549<br>2.981%	0.339<br>3.569%	0.416<br>3.969%	-0.267<br>5.006%
3s	0.390<br>2.972%	0.122<br>4.229%	0.881<br>1.529%	0.787<br>2.068%	0.786<br>2.126%	0.731<br>2.442%	0.623<br>3.014%	0.537<br>3.524%	0.436<br>3.870%
4s	-0.476<br>4.514%	0.306<br>4.192%	0.830<br>1.743%	0.704<br>2.440%	0.672<br>2.744%	0.532<br>3.449%	0.432<br>4.041%	0.319<br>4.663%	0.158<br>5.305%
5s	-0.641<br>4.674%	0.376<br>3.734%	0.853<br>1.578%	0.790<br>1.920%	0.750<br>2.153%	0.586<br>2.802%	0.520<br>3.548%	0.407<br>4.209%	0.229<br>5.074%

Table S4: Records of 6 different runs, each using different sizes of sliding windows, with randomly initialized CED model weights and random noisy data augmentation of the same transfer learning workflow. The results of the adopted sliding window size of 2s are provided in Table S3. The performance of the model trained in each run is measured by the coefficient of determination (R^2, top row in each cell) and the mean absolute percentage error (MAPE, bottom row in each cell, in %).

Model architecture	Training on FDEM	Direct predicting on Lab p4677	Transfer learning on Lab p4677	Predicting on Lab p4581 3MPa
Adopted design 4 layers, 8 filters (363,696)	-0.219 ± 0.228<br>4.050% ± 0.461%	0.200 ± 0.162<br>4.212% ± 0.424%	0.816 ± 0.0377<br>1.830% ± 0.163%	0.778 ± 0.0306<br>2.047% ± 0.145%
4 layers, 4 filters (91,416)	-0.307 ± 0.263<br>4.133% ± 0.297%	0.204 ± 0.0838<br>4.303% ± 0.414%	0.682 ± 0.123<br>2.635% ± 0.893%	0.506 ± 0.208<br>3.184% ± 0.895%
4 layers, 2 filters (23,100)	-0.0858 ± 0.189<br>3.702% ± 0.248%	-0.0131 ± 0.148<br>4.427% ± 0.187%	0.132 ± 0.317<br>3.626% ± 0.732%	0.267 ± 0.162<br>3.496% ± 0.289%
3 layers, 8 filters (95,536)	-0.175 ± 0.482<br>3.992% ± 0.764%	0.155 ± 0.252<br>4.479% ± 0.754%	0.836 ± 0.0346<br>1.787% ± 0.170%	0.764 ± 0.0343<br>2.150% ± 0.136%
2 layers, 8 filters (28,272)	-0.321 ± 0.463<br>4.146% ± 0.751%	0.266 ± 0.0246<br>4.248% ± 0.218%	0.840 ± 0.0505<br>1.816% ± 0.228%	0.704 ± 0.0811<br>2.110% ± 0.0820%

Table S5: Comparison of hyper-parameters of the model architecture (1) the number of convolutional filters , (2) the number of layers in the encoder and decoder. The numbers in parentheses indicate the number of trainable parameters in each model. The performance of the model trained in each run is measured by the coefficient of determination (R^2, first number in each cell) and the mean absolute percentage error (MAPE, second number in each cell, in %). Each model are run 5 times to get the mean and standard deviations of the measures.
